# Sigma-1 Receptor Agonism Promotes Mechanical Allodynia After Priming the Nociceptive System with Capsaicin

**DOI:** 10.1038/srep37835

**Published:** 2016-11-25

**Authors:** J. M. Entrena, C. Sánchez-Fernández, F. R. Nieto, R. González-Cano, S. Yeste, E. J. Cobos, J. M. Baeyens

**Affiliations:** 1 Institute of Neuroscience, Biomedical Research Center, University of Granada, 18100 Armilla, Granada, Spain; 2 Animal Behavior Research Unit, Scientific Instrumentation Center, University of Granada, 18100 Armilla, Granada, Spain; 3 Biosanitary Research Institute, University Hospital Complex of Granada, 18012 Granada, Spain; 4 Department of Pharmacology, School of Medicine, University of Granada, 18016 Granada, Spain; 5 Drug Discovery and Preclinical Development, Esteve, 08041, Barcelona, Spain; 6 Teófilo Hernando Institute for Drug Discovery, 28029 Madrid, Spain

## Abstract

Sigma-1 receptor antagonists promote antinociception in several models of pain, but the effects of sigma-1 agonists on nociception (particularly when the nociceptive system is primed) are not so well characterized; therefore we evaluated the effects of sigma-1 agonists on pain under different experimental conditions. The systemic administration of the selective sigma-1 agonists (+)-pentazocine and PRE-084, as well as the nonselective sigma-1 agonist carbetapentane (used clinically as an antitussive drug), did not alter sensitivity to mechanical stimulation under baseline conditions. However, they greatly promoted secondary mechanical allodynia after priming the nociceptive system with capsaicin. These effects of sigma-1 agonists were consistent in terms potency with the affinities of these drugs for sigma-1 receptors, were reversed by sigma-1 antagonists, and were not observed in sigma-1 knockout mice, indicating that they are sigma-1-mediated. Repeated systemic treatment with PRE-084 induced proallodynic effects even 24 h after treatment completion, but only after the nociceptive system was primed. However, neither the presence of this drug in the organism nor changes in sigma-1 receptor expression in areas involved in pain processing explains its long-term effects, suggesting that sustained sigma-1 agonism induces plastic changes in the nociceptive system that promote nociception.

The sigma-1 receptor is a ligand-regulated molecular chaperone that modulates several receptors and ion channels^1^,^2^,^3^ and is involved in a variety of neurological disorders^4^,^5^,^6^. Consequently, in recent years this pharmacological target has been the subject of intense basic research and drug development of both σ_1_ agonists and antagonists^5^,^7^. Selective σ_1_ ligands are available to study σ_1_ receptor function, including the prototypical σ_1_ agonists (+)-pentazocine and PRE-084, and the σ_1_ antagonists BD-1063, BD-1047 and S1RA^2^,^8^. In addition to these selective drugs, σ_1_ receptors bind a broad catalogue of compounds of very different structural classes and with different therapeutic and pharmacological applications. For example, the muscarinic antagonist carbetapentane, a widely used antitussive drug^9^, is a known σ_1_ agonist (see refs 2 and 10 for references), whereas the dopaminergic antagonist haloperidol, one of the most commonly prescribed antipsychotics^11^,^12^, is a well documented σ_1_ antagonist (see refs 2 and 10 for references).

Sigma-1 receptors are expressed in important areas for pain control within the central and the peripheral nervous system. In the central nervous system, these receptors are located in the superficial layers of the spinal cord dorsal horn^13^, and in supraspinal sites such as the periaqueductal gray matter and rostroventral medulla^3^,^14^. In the peripheral nervous system, these receptors are abundant in the dorsal root ganglion (DRG)^14^, specifically in the somas of peripheral sensory neurons^15^. Although σ_1_ antagonists do not alter mechanical or thermal thresholds under normal conditions (e.g. refs 8, 16 and 17), they are able to decrease the pain responses induced by chemical irritants such as formalin or capsaicin (e.g. refs 18, 19, 20) as well as the sensory gain (allodynia or hyperalgesia) occurring due to sensitization of the nociceptive system by capsaicin^8^,^16^,^21^ or by pathological states such as neuropathy, inflammation or ischemic pain^8^,^17^,^22^,^23^,^24^.

The analgesic potential of σ_1_ antagonists has thus been well documented, and in fact one σ_1_ antagonist (S1RA) is currently in phase II clinical trials^25^. However, the effects of σ_1_ agonists are controversial. Some studies showed that the administration of selective σ_1_ agonists such as PRE-084 or (+)-pentazocine had the opposite effects to σ_1_ antagonists, i.e., σ_1_ agonism was able to induce or promote pain hypersensitivity^23^,^26^,^27^,^28^,^29^,^30^,^31^. Other studies, however, showed that the administration of these compounds did not alter sensory thresholds at all^16^,^17^,^21^,^32^,^33^. Whether σ_1_ agonists are pronociceptive is not a trivial question, since as noted above, several drugs that are marketed for clinical use, although not selective for σ_1_ receptors, are able to bind this receptor and thereby act as nonselective σ_1_ agonists. Moreover, some selective σ_1_ receptor agonists are currently being tested in clinical trials^5^.

Therefore, to clarify whether σ_1_ activation is able to enhance pain sensitivity, we studied the effects of the acute administration of selective and nonselective σ_1_ agonists in different experimental pain conditions. We first tested whether σ_1_ agonists are able to increase acute pain induced by the intraplantar administration of capsaicin. In addition, because the σ_1_ receptor is known to play a role in pain sensitization, we tested whether σ_1_ agonism alone is able to induce sensitization to mechanical stimuli or to promote mechanical hypersensitivity after the administration of capsaicin to prime the nociceptive system.

It was previously reported that σ_1_ receptor expression can increase in the spinal cord^26^ and in peripheral nervous tissues^23^ in chronic pain models, and this might influence the development of sensory hypersensitivity^13^,^23^. Therefore, we also aimed to test whether chronic σ_1_ activation by the selective σ_1_ agonist PRE-084 is able to induce sensitization to mechanical stimuli *per se* or to promote mechanical hypersensitivity after the administration of capsaicin, and whether its effects are due to the altered expression of σ_1_ receptors in key areas of the pain pathways in the central and peripheral nervous system.

## Results

### Acute nociceptive behaviors and secondary mechanical allodynia induced by the intraplantar administration of capsaicin to wild-type mice

**Figure 1:**
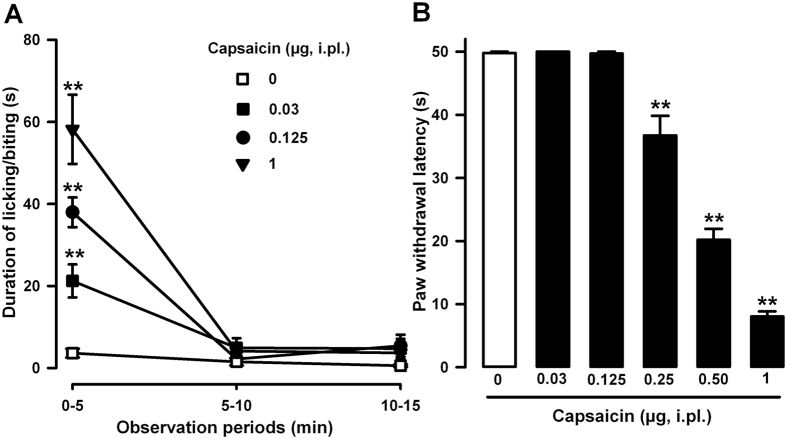
Behavioral effects induced by the intraplantar (i.pl.) administration of capsaicin (0.03–1.00 μg) in wild-type mice. (**A**) Time course (0–15 min) of acute pain behaviors (duration of licking or biting) after the administration of capsaicin or its vehicle (DMSO 1%, dose 0). (**B**) Latency to paw withdrawal in response to the application of a punctate mechanical stimulus at 0.5 g force 15 min after the i.pl. administration of capsaicin or its vehicle. Animals were always stimulated in the injected paw. (**A** and **B**) Each point or bar and vertical line represents the mean ± SEM of values obtained in 8–10 animals. Statistically significant differences between the values obtained in capsaicin- and vehicle-treated animals: **p < 0.01 (two-way repeated measures ANOVA for A, and one-way ANOVA for B; the Bonferroni post hoc test was used for all comparisons).

The intraplantar injection of capsaicin (0.03–1 μg, i.pl.) to wild-type mice evoked intense and dose-dependent nociceptive responses (licking and biting of the injected paw). These behavioral responses appeared immediately and peaked during the first 5 min after capsaicin administration. However, mice did not show appreciable nociceptive behaviors between 5 and 15 min after the i.pl. injection of capsaicin at any dose tested (Fig. 1A). Mice given the capsaicin solvent (dimethylsulfoxide, DMSO 1%) via i.pl. injection did not show overt pain-like responses in any observation period tested (dose 0 in Fig. 1A).

We also tested the ability of different doses of capsaicin to induce secondary mechanical allodynia. Hypersensitivity to mechanical stimulation was tested 15 min after capsaicin administration, when no observable spontaneous nociceptive behavior was detected. Animals given the capsaicin vehicle via i.pl. injection and stimulated in the injected paw with a light punctate mechanical stimulus (0.5 g force) showed values of paw withdrawal latency close to the cut-off time (50s) (Fig. 1B, white bar). However, mice treated with capsaicin (0.03–1 μg, i.pl.) showed a marked, dose-dependent reduction in their response latencies to mechanical stimulation, which was maximal with 1 μg capsaicin. It is worth noting a dose of capsaicin of 0.125 μg was sufficient to evoke overt acute nociceptive responses under our experimental conditions (Fig. 1A), without sensitizing the animals to the mechanical stimulus (Fig. 1B).

Therefore, the i.pl. administration of capsaicin was able to induce short-lasting but robust pain-like responses followed by mechanical hypersensitivity.

### Effects of the acute administration of σ_1_ agonists on nociceptive behaviors induced by the intraplantar administration of capsaicin

**Figure 2:**
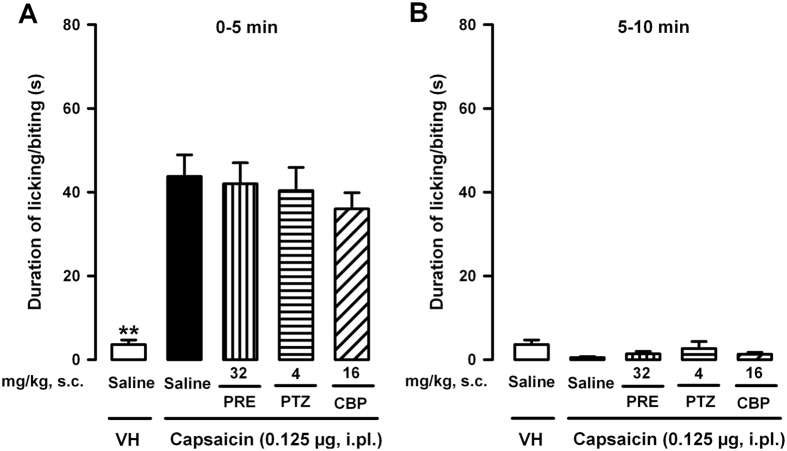
Effects of σ_1_ agonists on acute nociceptive behaviors induced by the intraplantar (i.pl.) administration of capsaicin in wild-type mice. The results show the duration of licking or biting of the paw injected with capsaicin (0.125 μg) or its vehicle (VH, DMSO 1%). Behavioral responses were recorded at intervals of 0–5 min (**A**) and 5–10 min (**B**) after the i.pl. administration of capsaicin or its vehicle. PRE-084 (PRE), (+)-pentazocine (PTZ), carbetapentane (CBP) or their solvent (saline) were subcutaneously (s.c.) administered 30 min before the i.pl. injection. (**A** and **B**) Each bar and vertical line represents the mean ± SEM of the values obtained in 8–10 animals. Statistically significant differences between the values obtained in mice treated with capsaicin or its solvent: **p < 0.01. There were no statistically significant differences between the values obtained in capsaicin-treated animals administered with the σ_1_ agonists or their solvent (two-way ANOVA).

We evaluated the effects of the s.c. administration of σ_1_ agonists on acute nociceptive behaviors induced by the injection of capsaicin 0.125 μg to wild-type mice. This dose of capsaicin evoked an intermediate duration of licking/biting (see Fig. 1A) that allowed us to identify potential increases or decreases induced by the σ_1_ agonists. As expected, in mice that were given capsaicin 0.125 μg (i.pl.) and the drug solvent (saline, s.c.), we observed clear nociceptive responses during the first 5 min after the administration of the chemical algogen, in comparison to the near absence of pain-like responses in mice treated with the solvents of capsaicin and the drugs tested (Fig. 2A). The s.c. administration of the selective σ_1_ agonists PRE-084 (32 mg/kg) or (+)-pentazocine (4 mg/kg) did not alter the acute pain-like responses induced by capsaicin during the initial 5 min after capsaicin administration (Fig. 2A). Similar results were seen in mice treated s.c. with the nonselective σ_1_ agonist carbetapentane (16 mg/kg) (Fig. 2A).

We also tested whether the s.c. administration of σ_1_ agonists was able to increase the duration (rather than the intensity) of the nociceptive behaviors induced by the i.pl. administration of capsaicin, by observing pain-like responses from 5 to 10 min after capsaicin injection. However, we detected no appreciable nociceptive behaviors in mice that were given the capsaicin solvent (as expected) or capsaicin alone or associated with any of the σ_1_ agonists tested (Fig. 2B).

Therefore, pharmacological σ_1_ agonism did not enhance the intensity or duration of acute pain-like responses induced by the i.pl. injection of capsaicin.

### Effects of the acute administration of σ_1_ agonists on mechanical sensitivity with or without priming of the nociceptive system with capsaicin

**Figure 3:**
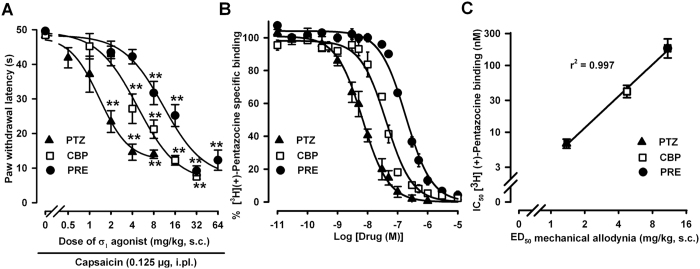
Comparison of the effects of σ_1_ agonists on capsaicin-induced secondary mechanical allodynia in wild-type mice, and affinity of these σ_1_ agonists for σ_1_ receptors labeled with [^3^H](+)-pentazocine. (**A**) Mice were subcutaneously (s.c.) injected with PRE-084 (PRE), (+)-pentazocine (PTZ), carbetapentane (CBP) or their solvent (saline, dose 0) 30 min before the intraplantar (i.pl.) administration of capsaicin (0.125 μg). The results show the latency to paw withdrawal in response to a punctate mechanical stimulus (0.5 g force) applied 15 min after the i.pl. administration of capsaicin. Animals were always stimulated in the injected paw. Each point and vertical line represents the mean ± SEM of the values obtained in 8–10 animals. Statistically significant differences between the values obtained in solvent-treated (dose 0) and drug-treated groups: **p < 0.01 (one-way ANOVA followed by Bonferroni test). (**B**) [^3^H](+)-pentazocine (5 nM) was incubated with 0.8 mg/mL brain membrane protein (P_2_ fraction) at 30 °C, pH 8, for 240 min and increasing concentrations of PTZ, CBP or PRE. Each point and vertical line represents the average of three experiments done in triplicate. (**C**) Correlation between the ED_50_ (dose of drug that produced half of the maximal decrease in paw withdrawal latency) and the IC_50_ (concentration of unlabeled drug that inhibited 50% of [^3^H](+)-pentazocine-specific binding) of the σ_1_ agonists tested.

We evaluated the effects of s.c. administration of σ_1_ agonists on mechanical sensitivity in animals treated with a low dose of capsaicin (0.125 μg), which as noted above does not induce sensitization to the mechanical stimulus (see Fig. 1B). The s.c. administration of the selective σ_1_ agonists PRE-084 (2–64 mg/kg) and (+)-pentazocine (0.5–8 mg/kg) to wild-type mice induced a dose-dependent decrease in the paw withdrawal latency in animals subjected to a light (0.5 g force) punctate mechanical stimulus on the capsaicin-injected paw (Fig. 3A). Similarly, the s.c. administration of carbetapentane (1–32 mg/kg) also dose-dependently potentiated the sensitizing effect of capsaicin to the mechanical stimulus when the mice were stimulated in the paw injected with capsaicin (Fig. 3A). Therefore, all three σ_1_ agonists evaluated here were able to facilitate allodynia to mechanical stimuli induced by capsaicin.

We also tested the affinities of PRE-084, (+)-pentazocine and carbetapentane for σ_1_ receptors in competition binding assays in wild-type mouse brain membranes, by labeling σ_1_ receptors with the selective σ_1_ radioligand [^3^H](+)-pentazocine. As shown in Fig. 3B, the specific binding of [^3^H](+)-pentazocine was concentration-dependently inhibited by the unlabeled σ_1_ agonists tested here.

Interestingly, the order of potency of these three σ_1_ receptor agonists in the potentiation of capsaicin-induced mechanical allodynia as shown by the dose of drug that produced half of its maximal effect (ED_50_ 1.39 ± 0.22 mg/kg for (+)-pentazocine, 4.85 ± 1.01 mg/kg for carbetapentane and 11.53 ± 3.66 mg/kg for PRE-084) parallels their affinity for σ_1_ receptors, measured as the concentration of unlabeled drug that inhibited 50% of [^3^H](+)-pentazocine-specific binding (IC_50_ 6.74 ± 0.37 nM for (+)-pentazocine, 42.14 ± 6.11 nM for carbetapentane and 187.71 ± 18.83 nM for PRE-084). In fact, we found a significant correlation between both variables (Fig. 3C).

**Figure 4:**
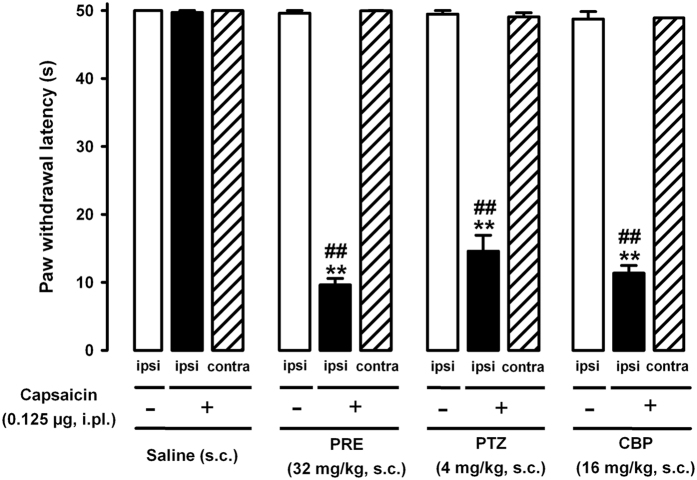
Comparison of the effect of σ_1_ agonists on capsaicin-sensitized and on nonsensitized wild-type mice. Mice were subcutaneously (s.c.) treated with PRE-084 (PRE), (+)-pentazocine (PTZ), carbetapentane (CBP) or their solvent (saline) 30 min before the intraplantar (i.pl.) injection of capsaicin (0.125 μg) or its vehicle (DMSO 1%). The results show latency to paw withdrawal in response to a mechanical stimulus (0.5 g) applied in the hindpaw. Capsaicin-sensitized mice were stimulated in both the ipsilateral (ipsi, black bars) and contralateral (contra, hatched bars) paw to the i.pl. injection. The response in the ipsilateral paw of nonsensitized (DMSO-treated) animals (ipsi, open bars) was also evaluated. Each bar and vertical line represents the mean ± SEM of the values obtained in 8–10 animals. Statistically significant differences between the values obtained in mice treated with the σ_1_ agonists or its solvent and stimulated in the paw ipsilateral to the capsaicin injection: **p < 0.01; and between the values obtained in mice stimulated in the ipsilateral or contralateral paw after the administration of capsaicin in the ipsilateral paw: ^##^*p* < 0.01. There were no statistically significant differences between the groups treated s.c. with saline (administered with capsaicin or its solvent), or between any of these three groups and mice treated with the σ_1_ agonists and stimulated in the paw contralateral to capsaicin administration. There were no statistically significant differences between the groups treated s.c. with saline and animals treated with the σ_1_ agonists and stimulated in the paw in which the capsaicin solvent was injected (two-way ANOVA followed by Bonferroni test).

We also tested whether doses of the σ_1_ agonists able to clearly potentiate capsaicin-induced mechanical allodynia were sufficient to induce sensitization to the mechanical stimulus in the absence of capsaicin administration. We found that the s.c. administration of PRE-084 (32 mg/kg), (+)-pentazocine (4 mg/kg) or carbetapentane (16 mg/kg), although it induced a clear decrease in the paw withdrawal latency of mice stimulated in the paw injected with 0.125 μg capsaicin (Fig. 4, black bars), did not decrease the latency to response in mice that were given the capsaicin vehicle and stimulated in the injected paw (Fig. 4, open bars). In addition, none of the σ_1_ agonists tested was able to decrease the paw withdrawal latency of capsaicin-injected mice when mechanical stimulation was applied to the contralateral paw (Fig. 4, hatched bars).

Therefore, the systemic administration of the selective σ_1_ agonists PRE-084 or (+)-pentazocine, as well as the nonselective σ_1_ agonist carbetapentane, was unable to sensitize mice to the mechanical stimulus *per se* but enhanced the mechanical hypersensitivity induced by a low dose of capsaicin, and this effect was only seen when the mice were stimulated in the paw injected with capsaicin and not when sensitivity to mechanical stimuli was tested at a distant site (contralateral paw).

### Role of σ_1_ receptor activation in the ability of σ_1_ agonists to potentiate capsaicin-induced mechanical allodynia

**Figure 5:**
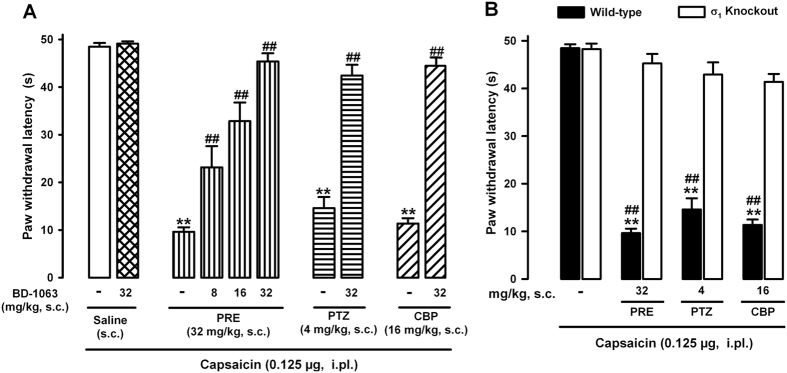
Specificity of the effects induced by σ_1_ agonists on capsaicin-induced secondary mechanical allodynia. (**A**) Reversion of the effects of σ_1_ agonists on capsaicin-induced secondary mechanical allodynia by the σ_1_ antagonist BD-1063 in wild-type mice. Wild-type mice were subcutaneously (s.c.) treated with BD-1063 (8–32 mg/kg) or its solvent (saline) alone or associated with PRE-084 (PRE, 32 mg/kg), (+)-pentazocine (PTZ, 4 mg/kg) or carbetapentane (CBP, 16 mg/kg). (**B**) Comparison of the proallodynic effects of σ_1_ agonists in wild-type and σ_1_ knockout mice. (**A** and **B**) The drugs tested were administered 30 min before the intraplantar (i.pl.) administration of capsaicin (0.125 μg). The results represent latency to paw withdrawal in response to stimulation with a punctate mechanical stimulus at 0.5 g force 15 min after capsaicin administration. Animals were always stimulated in the injected paw. Each bar and vertical line represents the mean ± SEM of the values obtained in 8–10 animals. (**A** and **B)** Statistically significant differences between wild-type mice treated with each σ_1_ agonist and mice treated with solvent: **p < 0.01. (**A**) Statistically significant differences between mice treated with each σ_1_ agonist alone or associated with BD-1063: ^##^*p* < 0.01. (**B**) Statistically significant differences between wild-type and σ_1_ knockout mice treated with σ_1_ agonists: ^##^*p* < 0.01. There were no statistically significant differences between (**A**) the values in wild-type mice treated with BD-1063 alone or its solvent, and (**B**) between σ_1_ knockout mice treated with the σ_1_ agonists or their solvent (two-way ANOVA followed by Bonferroni test).

To test for the selectivity of the effects induced by PRE-084, (+)-pentazocine and carbetapentane on capsaicin-induced secondary mechanical allodynia, we evaluated the effects of the association of the s.c. administration of these σ_1_ agonists with several σ_1_ antagonists in wild-type mice. We found that in contradistinction to the strong potentiation of capsaicin-induced mechanical allodynia by the σ_1_ agonists PRE-084, (+)-pentazocine or carbetapentane, the s.c. administration of the selective σ_1_ antagonist BD-1063 (32 mg/kg) was unable to decrease latency to the behavioral response induced by the mechanical stimuli in mice that were given capsaicin 0.125 μg (Fig. 5A). However, when BD-1063 (8–32 mg/kg, s.c.) was associated with the selective σ_1_ agonist PRE-084 (32 mg/kg, s.c.), the σ_1_ antagonist induced a dose-dependent, complete reversion of the effects induced by PRE-084, increasing the paw withdrawal latency to values similar to those seen in mice treated with the drug solvent. Similarly, the s.c. administration of BD-1063 (32 mg/kg) also abolished the effects of (+)-pentazocine (4 mg/kg, s.c.) and carbetapentane (16 mg/kg, s.c.) on capsaicin-induced allodynia (Fig. 5A).

To ensure that the reversion of the effects of σ_1_ agonists was not a peculiarity of BD-1063, we tested whether potentiation of capsaicin-induced allodynia by the selective σ_1_ agonist PRE-084 could also be reversed by a variety of σ_1_ antagonists. As in the results for the effects of BD-1063, we found that the administration of the selective σ_1_ antagonists BD-1047 (64 mg/kg, s.c.) and S1RA (64 mg/kg, s.c.), as well as the nonselective σ_1_ antagonist haloperidol (0.06 mg/kg, s.c.), fully reversed the effect of PRE-084 (32 mg/kg, s.c.) on capsaicin-induced mechanical allodynia (Supplementary Fig. 1).

Because haloperidol is a known dopaminergic antagonist, we controlled for the influence of dopaminergic antagonism on the effects we observed by testing (−)-sulpiride, a dopaminergic antagonist lacking affinity for σ_1_ receptors^34^,^35^. The s.c. administration of this drug alone (200 mg/kg) did not modify paw withdrawal latency in animals injected with the low dose of capsaicin, and its association with PRE-084 (32 mg/kg, s.c.) did not alter its effect on capsaicin-induced mechanical hypersensitivity (Supplementary Fig. 1), suggesting that dopaminergic activity did not account for the effects. We previously verified that the solvent of haloperidol and (−)-sulpiride (5% gum arabic) did not alter the responses to mechanical stimuli in mice treated with capsaicin or its vehicle (data not shown).

Therefore, the s.c. administration of σ_1_ antagonists fully reversed the potentiation of the σ_1_ agonists tested here on capsaicin-induced mechanical allodynia.

To further verify the selectivity of the effects of σ_1_ agonists, we tested their effects on mice lacking σ_1_ receptors. Wild-type and σ_1_ knockout mice (σ_1_ KO) showed similar responses when stimulated in the paw injected with capsaicin 0.125 μg. However, although wild-type mice given the σ_1_ agonists PRE-084 (32 mg/kg, s.c.), (+)-pentazocine (4 mg/kg, s.c.) or carbetapentane (16 mg/kg, s.c.) showed strong potentiation of capsaicin-induced secondary mechanical allodynia, the behavioral response to mechanical stimuli in σ_1_ knockout mice in which capsaicin was injected was not significantly changed by the administration of any of these σ_1_ agonists (Fig. 5B).

The absence of activity of PRE-084, (+)-pentazocine and carbetapentane in mice lacking σ_1_ receptors suggests that off-target effects do not contribute to the potentiation of capsaicin-induced allodynia by these drugs.

### Effects of repeated treatment with PRE-084 on capsaicin-induced secondary mechanical allodynia

We also tested the effects of chronic σ_1_ activation on capsaicin-induced secondary mechanical allodynia. For these assays we administered PRE-084 (32 mg/kg, s.c.) once a day during 7 days. To minimize the presence of PRE-084 in the organism during behavioral testing, we did the experiments 24 h after the last s.c. administration. This allows us to explore whether chronic σ_1_ activation is able to promote a long lasting sensitization to mechanical stimulus in response to capsaicin, even after the expected clearance of the drug.

**Figure 6:**
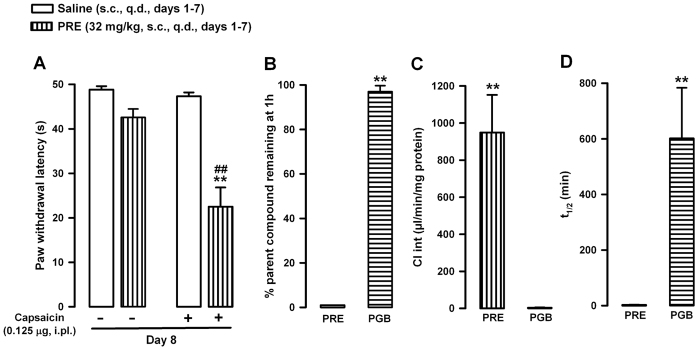
Effect of the repeated administration of PRE-084 (PRE) on capsaicin-sensitized mice and on nonsensitized animals, and the *in vitro* metabolic stability of PRE and pregabalin (PGB) in mouse liver microsomes. (**A**) Mice were subcutaneously (s.c.) injected with PRE 32 mg/kg or its solvent (saline) once a day (q.d.) for 7 consecutive days, and were tested 24 h after the last s.c. administration. The results represent latency to paw withdrawal in response to stimulation with a punctate mechanical stimulus (0.5 g force) 15 min after the intraplantar (i.pl.) administration of capsaicin (0.125 μg) or its vehicle (DMSO 1%). Animals were always stimulated in the injected paw. Each bar and vertical line represents the mean ± SEM of the values obtained in 8–10 animals. Statistically significant differences between the values from mice treated i.pl. with capsaicin and repeatedly treated with PRE or its solvent: **p < 0.01. Statistically significant differences between mice repeatedly treated with PRE and treated i.pl. with capsaicin or its vehicle: ^##^*p* < 0.01. There were no significant differences between mice treated i.pl. with capsaicin vehicle and repeatedly treated with PRE or its solvent (two-way ANOVA followed by Bonferroni test). (**B, C** and **D**) Indicators of the *in vitro* metabolic stability of PRE-084 and PGB: (**A**) percent remaining drug after incubation for 1 h, (**B**) intrinsic clearance of the drug (Cl _int_), and (**C**) metabolic half-life (t_1/2_). The concentration of compounds was determined by ultra-high performance liquid chromatography and tandem mass spectrometry after incubation in mouse liver microsomes. Each bar and vertical line represents the mean ± SEM of values obtained in 3 samples. Statistically significant differences between the values obtained after incubation with PRE or PGB in mouse liver microsomes: **p < 0.01 (Student’s *t* test).

In nonsensitized animals (capsaicin vehicle administered i.pl.), the repeated administration of the σ_1_ agonist did not significantly modify paw withdrawal latency to mechanical stimuli in comparison to saline-treated controls (Fig. 6A). However, repeated treatment with PRE-084 enhanced the sensitivity to mechanical stimuli induced by capsaicin even 24 h after the last administration of the σ_1_ agonist, markedly decreasing paw withdrawal latency in mice treated with capsaicin in comparison to animals treated with capsaicin and given repeated doses of saline (Fig. 6A).

These results indicate that repeated systemic σ_1_ agonism is not sufficient in itself to induce sensitization to the mechanical stimulus, but it is able to enhance capsaicin-induced mechanical hypersensitivity in a sustained manner.

Our results might have been due to the low clearance of PRE-084, since during the timeframe of our assays, the concentration of this drug in the organism might remain high to potentiate capsaicin. To rule out this possibility we tested the rate of PRE-084 metabolism on mouse liver microsomes, using pregabalin (PGB) as a control for a drug known to be poorly metabolized in the liver^36^. After incubating mouse liver microsomes for 1 h with PRE-084, we were unable to detect any remaining drug in the incubation media, due to the high intrinsic clearance (Cl int) and consequently very short half-life (t_1/2_) (see Fig. 6B,C and D). This result indicates that PRE-084 undergoes rapid, extensive liver metabolism, and suggests that the *in vivo* effects of repeated treatment with PRE-084 are unlikely to be attributable to the presence of this drug in the organism at the time of behavioral testing (24 h after the last administration). In contrast, the control drug PGB showed high metabolic stability in mouse liver microsomes, with values close to the 100% of the initial concentration after incubation for 1 h, due to a low Cl int and a very long t_1/2_ (Fig. 6B,C and D), as expected. Both drugs produced similar results in mouse and human liver microsomes, with PRE-084 being rapidly metabolized and PGB being slowly metabolized in both species (Supplementary Table 1).

**Figure 7:**
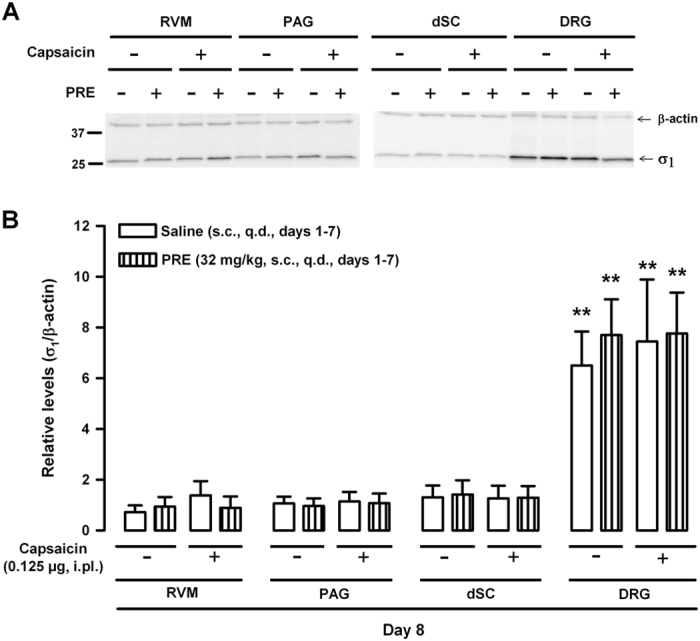
Expression of σ_1_ receptors in the rostroventral medulla (RVM), periaqueductal grey matter (PAG), dorsal spinal cord (dSC) and dorsal root ganglion (DRG) after the repeated administration of PRE-084 (PRE) in capsaicin-sensitized mice and nonsensitized animals. Mice were subcutaneously (s.c.) treated with PRE or its solvent (saline) once a day (q.d.) for 7 days. 24 h after the last s.c. injection, the mice were given an intraplantar injection of capsaicin (0.125 μg) or its vehicle (DMSO, 1%), and tissue samples were obtained 15 min later. (**A**) Representative immunoblots for σ_1_ receptors in mice after different treatments. β-actin was used as a loading control. Full-length gels are presented in Supplementary Figure 2. (**B**) Quantification of immunoblotting for σ_1_ receptors under different experimental conditions. Each bar and vertical line represents the mean ± SEM of densitometric values obtained in 4–5 animals. The result for σ_1_ receptor band intensities were expressed relative to those of their corresponding β-actin loading control bands. Statistically significant differences between samples from central nervous system regions (RVM, PAG, dSC) and lumbar DRGs from mice with the same treatment: **p < 0.01. There were no significant differences between mice treated with PRE or its solvent, or between animals treated with capsaicin or its vehicle, in any tissue tested (two-way ANOVA followed by Bonferroni test). All samples derive from the same experiments and gels were processed in parallel.

To test whether the potentiation of capsaicin-induced mechanical hypersensitivity by repeated treatment with PRE-084 was accompanied by changes in the expression of σ_1_ receptors in key areas of the pain pathways, we carried out western blotting of tissue samples from central sites, including the dorsal spinal cord (dSC) and supraspinal areas such as the rostroventral medulla (RVM) and the periaqueductal grey matter (PAG), as well as from peripheral nervous system locations such as the DRG. We found that σ_1_ receptors were present in all areas of the nervous system examined here, and were expressed more prominently in the DRG than in central areas (Fig. 7A and B). The repeated systemic administration of PRE-084 did not change the expression of σ_1_ receptors in any area examined, in mice that were given capsaicin or its vehicle (Fig. 7A and B). Therefore, the behavioral effects of repeated treatment with PRE-084 cannot be explained by alterations in the expression of σ_1_ receptors.

## Discussion

We found that the acute systemic administration of σ_1_ agonists, including selective (PRE-084 and (+)-pentazocine) or nonselective (carbetapentane) drugs, although unable to modify acute pain induced by capsaicin or to induce mechanical allodynia by themselves, can greatly increase mechanical sensitivity after the nociceptive system is primed with a low dose of capsaicin. In addition, the repeated systemic administration of the selective σ_1_ agonist PRE-084 induced a long-lasting proallodynic effect, but this was detectable only after priming the nociceptive system.

We found that the intraplantar injection of capsaicin induced a dose-dependent increase in pain-related behaviors and delayed secondary hypersensitivity to mechanical stimulation, as previously reported^16^,^37^,^38^. Secondary hypersensitivity is produced in an area close to the capsaicin injection, and is known to be the result of the central sensitization due to the previous C-nociceptor barrage induced by capsaicin^39^. Importantly, the doses of capsaicin able to elicit overt pain responses are lower than those needed to induce secondary mechanical allodynia, as shown in this and previous studies (e.g. ref. 38). Therefore, a painful stimulus will not always lead to central sensitization, and an intensity threshold needs to be exceeded for secondary hypersensitivity to develop.

Here we show that a dose of capsaicin of 0.125 μg, although it induced obvious pain behaviors, was unable to trigger mechanical hypersensitivity. The acute systemic administration of the three σ_1_ agonists tested here (PRE-084, (+)-pentazocine and carbetapentane) clearly allowed this low dose of capsaicin to induce robust secondary mechanical hypersensitivity, but importantly, none of these σ_1_ agonists enhanced the intensity or duration of capsaicin-induced nociceptive behaviors. This indicates that the enhanced mechanical allodynia caused by the combination of capsaicin and σ_1_ agonism was not the result of an increase in early acute pain induced by the chemical algogen. Therefore, σ_1_ agonism might decrease the intensity threshold of acute pain needed to induce the secondary mechanical hypersensitivity, and this effect points to an enhancement of capsaicin-induced central sensitization as the underlying mechanism.

Several arguments support the specificity of the facilitatory effects of these σ_1_ agonists on capsaicin-induced mechanical allodynia. (1) Their order of potency to facilitate capsaicin-induced sensitization parallels their affinity for σ_1_ receptors. (2) The effects of the σ_1_ agonists can be reversed by known σ_1_ antagonists, including the selective drugs BD-1063, BD1047 and S1RA, and also the nonselective dopaminergic/σ_1_ antagonist haloperidol (but not by the dopaminergic antagonist (−)-sulpiride). (3) None of the three σ_1_ agonists was able to enhance capsaicin-induced allodynia in mice lacking σ_1_ receptors (σ_1_ knockout mice). All of these results support the involvement of on-target mechanisms in the effects of the σ_1_ agonists.

It was recently reported that the local administration of PRE-084 synergistically enhanced the mechanical allodynia induced by low pH (which activates acid-sensing ion channels) and α-β-methylene-ATP (an agonist of P2-purinoreceptors)^23^. These findings together with our results indicate that σ_1_ agonism is able to promote to the development of mechanical allodynia after priming of the nociceptive system by several agents.

We also show that the acute systemic administration of the selective σ_1_ agonists PRE-084 and (+)-pentazocine as well as the nonselective agonist carbetapentane, although markedly potentiating capsaicin-induced mechanical allodynia, was unable to induce mechanical hypersensitivity *per se*. These results are consistent with previous findings that selective σ_1_ agonists (such as PRE-084 or (+)-pentazocine) did not alter sensory thresholds when administered systemically^16^,^17^,^21^,^33^, supraspinally (e.g. ref. 32) or peripherally^17^,^23^, at doses able to reverse the effect of σ_1_ antagonists (i.e. able to interact with σ_1_ receptors). Interestingly, carbetapentane, which is marketed as an antitussive, does not – to our knowledge – induce pain or sensory hypersensitivity in humans as a side effect. Therefore, these findings together suggest that σ_1_ agonism alone is not enough to induce pain. However, it was previously reported that the intrathecal administration of σ_1_ agonists is sufficient to clearly trigger mechanical allodynia^13^,^28^,^30^,^31^. One explanation for the proalgesic effects of spinal σ_1_ agonism is that the procedure for intrathecal catheterization might prime the nociceptive system, thus facilitating the pronociceptive effect of σ_1_ agonism. It is worth noting that similar to the effects of σ_1_ agonists on mechanical hypersensitivity, these drugs do not alter thermal thresholds when administered either systemically, supraspinally, or peripherally (e.g. refs 17 and 32) but their i.t. administration was also able to induce thermal hyperalgesia^26^,^30^. Therefore, the pronociceptive effect of σ_1_ agonism appears to be extensive to other types of pain hypersensitivity in addition to mechanical allodynia.

We also evaluated the effects of chronic σ_1_ agonism by repeatedly administering PRE-084. As seen for the acute effects of σ_1_ agonism, chronic treatment with PRE-084 did not alter the responses to mechanical stimulation unless the nociceptive system was primed with capsaicin. Importantly, capsaicin-induced mechanical allodynia was potentiated as long as 24 h after the last dose of PRE-084. We show that this drug undergoes extensive and extremely fast metabolism in liver microsomes; therefore the presence of the drug in the organism 24 h after its last administration cannot explain the behavioral effects we observed. It was previously reported that σ_1_ receptor expression can increase in the spinal cord^13^ and in peripheral nervous tissues^23^ in chronic pain models, and that this might influence the development of sensory hypersensitivity^13^,^23^. Therefore, we tested whether the long-lasting enhancement of capsaicin-induced mechanical allodynia by chronic treatment with PRE-084 was due to altered expression of σ_1_ receptors in key areas of the pain pathways. However, we detected no apparent changes in σ_1_ receptor density in either central sites (RVM, PAG, dSC) or in peripheral tissues (DRG). Sigma-1 agonism is known to promote the translocation of neurotransmitter receptors such as *N*-methyl-D-aspartate receptors to the plasma membrane, with the consequent enhancement of their excitatory actions^40^. These plastic changes might outlast the presence of the drug in the organism, so the biological half-life of the drug’s effects may be longer than the half-life of the drug in the organism. Alternatively, it was recently described that σ_1_ receptors can produce genomic actions by acting as transcriptional regulators at the nuclear envelope^41^. Either of these processes could explain the long-lasting effects of chronic treatment with PRE-084 (without upregulation of σ_1_ receptors) despite the rapid metabolism of this drug.

Current research on σ_1_ receptors is intense, with several pharmaceutical companies and academic institutions interested in the potential clinical use of selective σ_1_ ligands for a variety of intended indications. Sigma-1 antagonists have been proposed as analgesics, antipsychotics and as treatments for drug abuse^2^,^3^,^5^,^42^, whereas σ_1_ agonists have been proposed as potentially useful for anxiety, depression, post-stroke recovery, traumatic brain injury, spinal cord injury, and neurodegenerative diseases such as Alzheimer’s disease, Parkinson’s disease and multiple sclerosis^2^,^5^,^7^,^42^. Currently, several recently developed selective σ_1_ ligands are undergoing clinical trials for several of these indications (reviewed by ref. 5). It is worth noting that in some of the pathological conditions for which σ_1_ agonists are being investigated, pain is one of the symptoms. Examples include recovery from stroke^43^, traumatic brain injury^44^ or spinal cord injury^45^, Alzheimer’s disease^46^, Parkinson’s disease^47^ and multiple sclerosis^48^. In addition, other diagnoses such as anxiety or depression often occur concomitantly to pain states^49^. Here we show that although selective σ_1_ agonists are not pronociceptive by themselves, they can enhance pain sensitivity after the nociceptive system is primed, and this effect lasts longer than the predicted presence of the drug in the organism. Therefore, further studies should be encouraged of the pain state in patients with these diseases or disorders in clinical trials with σ_1_ agonists.

In summary, we found that σ_1_ receptor agonism can promote pain hypersensitivity after priming the nociceptive system. These findings may have implications in terms of safety for the development of σ_1_ agonists.

## Materials and Methods

### Animals

Experiments were done in female wild-type (Charles River, Barcelona, Spain) and σ_1_ knockout CD-1 mice (Laboratorios Esteve, Barcelona, Spain) weighing 25 to 30 g. Mutant mice were generated on a CD-1 background as described previously^16^. The animals were housed and acclimated to our facilities for at least 4 days before testing. They were maintained in temperature- and light-controlled conditions (22 ± 1 °C, lights on at 08:00 and off at 20:00, air replacement every 20 min), and had free access to a standard laboratory diet (Harlan Teklad Research Diet, Madison, WI, USA) and tap water *ad libitum* until the beginning of the experiments. The experiments were done during the light phase (09:00–15:00 hours), and at random times throughout the estrous cycle. All experimental protocols were carried out in accordance with international standards (European Communities Council directive 2010/63), and were approved by the Research Ethics Committee of the University of Granada.

### Drugs and drug administration for behavioral assays

We used a variety of chemically unrelated σ_1_ ligands, including selective and nonselective σ_1_ agonists and antagonists. The selective σ_1_ agonists used were PRE-084 [2-(4-morpholinethyl)1-phenylcyclohexanecarboxylate) hydrochloride] (Tocris Cookson Ltd., Bristol, UK) and (+)-pentazocine (Sigma–Aldrich S.A., Madrid, Spain) (reviewed by ref. 2). We also tested the effect of the nonselective σ_1_ agonist carbetapentane (Sigma–Aldrich).

We also used the σ_1_ antagonists BD-1063 (1-[2-(3,4-dichlorophenyl) ethyl]-4-methylpiperazine dihydrochloride), BD-1047 (*N*-[2-(3,4-dichlorophenyl)ethyl]-*N*-methyl-2-(dimethylamino)ethylamine dihydrobromide) (both from Tocris Cookson), and S1RA (4-[2-[[5-methyl-1-(2-naphthalenyl)1*H*-pyrazol-3-yl]oxy]ethyl] morpholine hydrochloride) (Laboratorios Esteve, Barcelona, Spain). All these drugs are considered selective for σ_1_ receptors^2^,^8^,^50^,^51^. In addition, we also used the nonselective σ_1_ antagonist haloperidol^2^ and the dopaminergic antagonist (−)-sulpiride^52^ (both from Sigma–Aldrich S.A.). Whereas haloperidol is a known high-affinity antagonist for both dopamine and σ_1_ receptors^34^,^35^, (−)-sulpiride binds to dopamine receptors but not to σ_1_ receptors^35^,^52^,^53^.

All drugs were dissolved in sterile physiological saline (NaCl 0.9%) to their final concentrations immediately before administration, with the exception of haloperidol and (−)-sulpiride, which were suspended in 5% gum arabic (Sigma-Aldrich S.A.). Drugs were injected subcutaneously into the interscapular zone in a volume of 5 mL/kg. When σ_1_ antagonists were used to reverse the effects of the σ_1_ agonists, they were injected s.c. immediately before the other drug solution. Each drug was injected into a different area of the interscapular zone to avoid physicochemical interaction between the drug solutions that might interfere with the results. An equal volume of vehicle was used in control animals. The chemical algogen used was capsaicin (Sigma–Aldrich S.A.), which was dissolved in 1% dimethylsulfoxide (DMSO, Merck KGaA, Darmstadt, Germany) in physiological saline to a final concentration of 0.05 μg/μL. Further dilutions were prepared in 1% DMSO in physiological saline. Capsaicin solutions or their solvent were injected intraplantarly (i.pl.) proximate to the heel into the ventral surface of the right hind paw in a volume of 20 μL, using a 1710 TLL Hamilton microsyringe (Teknokroma, Barcelona, Spain) with a 30^1/2^-gauge needle. To test the acute effects of the drugs on either capsaicin-induced acute nociceptive responses or secondary mechanical allodynia, drugs were administered s.c. 30 min before the i.pl. injection. To test the effects of chronic σ_1_ activation, the animals were s.c. treated once a day (q.d.) during 7 consecutive days with PRE-084 (or its solvent), and capsaicin-induced secondary mechanical allodynia was evaluated 24 h after the last s.c. administration to minimize the presence of PRE-084 in the organism during behavioral testing.

### [^3^H](+)-Pentazocine binding assays

For competition binding assays, the radioligand used was [^3^H](+)-pentazocine, with a specific activity of 32.2 Ci/mmol (PerkinElmer Life Sciences, Boston, MA, USA). Experiments were performed in crude synaptosomal membranes (P_2_ fraction) from wild-type mouse brain, according to a previously described method^14^,^16^,^21^. Detailed methods are reported in the Supplementary Information.

### Evaluation of capsaicin-induced acute nociceptive-like behaviors

Nociceptive-like behaviors induced by the i.pl. injection of capsaicin were studied as previously described^37^, with slight modifications. The animals were placed for 1-h acclimation period in the experimental room before the experiments. After this period the mice were gently restrained and 20 μL of capsaicin solution or its solvent was injected into the right hindpaw. Immediately thereafter, the mice were put into a glass cylinder and the observation period started. A mirror was placed behind the cylinder to allow clear observation of the paws throughout the evaluation period. The time spent licking or biting the injected paw during 15 min (divided into three periods of 5 min each) was measured as an indicator of the pain response.

### Evaluation of capsaicin-induced secondary mechanical allodynia

Mechanical allodynia was assessed as previously described^16^,^54^. Briefly, mice were habituated in individual black-walled test compartments placed on an elevated mesh-bottomed platform for 2 h before the experiment. Then the animals were carefully removed from the compartment and given an i.pl. injection of capsaicin or its solvent. Fifteen minutes later, a Dynamic plantar Aesthesiometer (Ugo Basile, Comerio, Italy) was used to apply a fixed nonflexible metallic filament (0.5 mm diameter), which was electronically driven into the ventral side of the hindpaw at least 5 mm away from the site of injection towards the toes (area of secondary mechanical hypersensitivity). When the paw contralateral to the side of capsaicin administration was evaluated, it was stimulated in an equivalent area (distal to the heel). An electronically controlled intensity of 0.5 g force was used throughout the experiments. This intensity of the stimulus was previously reported to be innocuous and unable to induce paw withdrawal in nonsensitized mice^16^,^21^. When nocifensive paw withdrawal occurred, the stimulus was automatically terminated and the response latency was automatically recorded. Each mouse was tested in three trials at 30 s intervals in each experimental session. A cut-off time of 50 s was used in each trial. The mean of the 3 trial values was recorded as the animal’s response.

To minimize the stress induced by excessive handling of the mice that might alter the behavioral results, animals evaluated for capsaicin-induced mechanical allodynia were always different to those used to determine capsaicin-induced acute nociception.

### Western blotting

Possible changes in the expression of σ_1_ receptors in key areas of the pain pathways were studied by western blotting. These areas included the PAG, RVM and dSC of lumbar segments 3 and 4 (L3-L4), and DRGs in L3-L4. A mouse monoclonal antibody that recognized sigma-1 receptor (1:1,000, sc-137075, Santa Cruz Biotechnology, Dallas, TX, USA) was used in all experiments. The specificity of this antibody was previously documented in σ_1_ KO animals^14^. A mouse monoclonal anti-β-actin antibody (1:5,000, sc-81178, Santa Cruz Biotechnology) was used as a loading control. Detailed methods are reported in the Supplementary Information.

### *In vitro* metabolic stability in liver microsomes

Incubation of the test compounds in mouse or human liver microsomes (Xenotech, Lenexa, KS, USA) was done with a previously described method^55^ with modifications. We tested the metabolic stability of PRE-084 and PGB, the latter of which was used as a control because of its poor hepatic metabolism^36^. Briefly, the drugs were incubated in 96-well plates at a concentration of 1 μM and at 37 °C during 1 h under standard incubation conditions: sodium-potassium phosphate buffer (50 mM, pH 7.4), MgCl_2_ (3 mM), the NADPH-regenerating system and cytochrome P450 content (0.3 nmol/mL). Aliquots of the reaction mixture were obtained at 0, 10, 20, 40 and 60 min with an equal volume of cold acetonitrile. The assay was done in a robotic liquid handling system (Freedom Evo Tecan, Männedorf, Switzerland). Upon centrifugation of the resulting mixture, supernatants were analyzed with a generic ultra-high performance liquid chromatography–tandem mass spectrometry method.

### Data analysis

The data were analyzed with the SigmaPlot 12.0 program (Systat Software Inc., San Jose, CA, USA). The ED_50_ (dose of drug that produced half of the maximal decrease in paw withdrawal latency) of the σ_1_ agonists was calculated from the dose–response curves by nonlinear regression analysis of the equation for a sigmoid plot. For binding assays, the IC_50_ (concentration of unlabeled drug that inhibited 50% of [^3^H](+)-pentazocine-specific binding) was estimated from the inhibition curves by nonlinear regression analysis of the equation for a sigmoid plot, assuming 1-site competition.

For the *in vitro* metabolic stability in liver microsomes, Ln-linear plots of the percentage of compound remaining based on chromatographic peak area *versus* time were generated, and the slope was calculated by linear fitting of the curve. The *in vitro* metabolic t_1/2_ was estimated with the equation 0.693/k where k is the biotransformation rate constant and corresponds to the slope of the ln-linear curve. Liver microsome Cl int was calculated with the equation 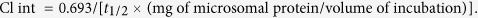


When the differences between the means of several experimental groups were compared, statistical analysis consisted of one-way or two-way analysis of variance (ANOVA) followed by the Bonferroni post hoc test in both cases. When the means of only two experimental groups were compared, we used Student’s *t* test. In all cases the differences between means were considered statistically significant when the value of *p* was below 0.05.

## Additional Information

**How to cite this article**: Entrena, J. M. *et al.* Sigma-1 Receptor Agonism Promotes Mechanical Allodynia After Priming the Nociceptive System with Capsaicin. *Sci. Rep.* **6**, 37835; doi: 10.1038/srep37835 (2016).

**Publisher's note:** Springer Nature remains neutral with regard to jurisdictional claims in published maps and institutional affiliations.

## Supplementary Material

Supplementary Information
