# Supplementary material for: Sigma-1 Receptor Agonism Promotes Mechanical Allodynia After Priming the Nociceptive System with Capsaicin
Source: Sci Rep. 2016 Nov 25;6:37835. doi: 10.1038/srep37835 (PMC5122889; doi:10.1038/srep37835)
Supplement: Supplementary Information [file srep37835-s1.pdf]

SUPPLEMENTARY INFORMATION

SIGMA-1 RECEPTOR AGONISM PROMOTES MECHANICAL ALLODYNIA AFTER PRIMING THE NOCICEPTIVE SYSTEM WITH CAPSAICIN

J.M. Entrena, C. Sánchez-Fernández, F.R. Nieto, R. González-Cano, S. Yeste, E.J. Cobos, J.M. Baeyens

Contents

1. **Supplementary materials and methods**

   1.1. [^{3}H](+)-Pentazocine binding assays

   1.2. Western blotting

2. **Supplementary figures and tables**

3. **References**

1. Supplementary materials and methods

1.1. [^{3}H](+)-Pentazocine binding assays

For competition binding assays, the radioligand used was [^{3}H](+)-pentazocine, with a specific activity of 32.2 Ci/mmol (PerkinElmer Life Sciences, Boston, MA, USA). Dilutions from the stock [^{3}H](+)-pentazocine solution were prepared with ice-cold incubation buffer (50 mM Tris–HCl buffer, pH 8 at 30°C). The cold drugs used were PRE-084, (+)-pentazocine and carbetapentane. PRE-084 and carbetapentane were dissolved in deionized ultrapure water, and (+)-pentazocine was dissolved in absolute ethanol to make up a stock solution of 1 mM. Further dilutions were prepared with incubation buffer to yield a final maximal concentration of ethanol in the incubation medium of 1% (v/v). We previously verified that this final concentration of ethanol did not affect the binding of [^{3}H](+)-pentazocine (1).

Competition binding assays were performed in crude synaptosomal membranes (P_2 fraction) from wild-type mouse brain, according to a previously described method (2-4). Briefly, brain membrane solutions (460 µL) were incubated at a final protein concentration of 0.8 mg/mL with 20 µL of several concentrations of the cold drug (from 10^{-11} to 10^{-5} M) or its solvent and 20 µL of [^{3}H](+)-pentazocine (final concentration of 5 nM) for 240 min at 30°C and pH 8. Five milliliters of ice-cold filtration buffer (Tris 10 mM, pH 7.4) was added to the tubes, and the solutions were immediately filtered (Brandel cell harvester model M-12 T; Brandel Instruments, SEMAT Technical, St. Albans, Hertfordshire, UK) over Whatman GF/B glass fiber filters (SEMAT Technical) presoaked with 0.5% polyethylenimine in Tris 10 mM, pH 7.4. The filters were washed twice with 5 mL ice-cold filtration buffer. Liquid scintillation cocktail (4 mL; CytoScint scintillation counting solution; MP Biomedicals, Irvine, CA, USA) was added to each filter and on the next day, radioactivity level was measured by liquid scintillation spectrometry (Beckman Coulter España SA, Madrid, Spain). The specific binding of (+)-pentazocine always represented more than 80% of the total binding.

1.2. Western blotting

Possible changes in the expression of σ_1 receptors in key areas of the pain pathways were assessed by western blotting. These areas included the periaqueductal grey matter (PAG), rostroventral medulla (RVM), dorsal spinal cord of the lumbar (L3-L4) enlargement (dSC) and lumbar (L3-L4) dorsal root ganglia (DRG). They were carefully removed from wild-type animals repeatedly treated s.c. with PRE-084 or saline, the day after the last subcutaneous (s.c.) administration and 15 min after the intraplantar (i.pl.) injection of capsaicin or its vehicle. The tissue was homogenized by sonication in a buffer solution (50 mM Tris, 150 mM NaCl, 2 mM EDTA, 0.5% Triton X-100, 0.1%

SDS, 1% NP-40, 1 mM phenylmethylsulfonyl fluoride, 1 mM Na_3VO_4, 25 mM NaF, 1% phosphatase inhibitor cocktail (P5726, from Sigma-Aldrich Quimica, Madrid, Spain), 0.5% protease inhibitor cocktail (P8340, from Sigma-Aldrich Quimica). Protein concentrations in the tissue homogenates were measured with the Bradford method, and the samples were stored at −80°C until use.

Twenty micrograms of protein was loaded on 12% (wt/vol) SDS-polyacrylamide gels and transferred electrophoretically to nitrocellulose membranes (Bio-Rad, Madrid, Spain). The membranes were blocked at room temperature for 1 h with blocking buffer containing 5% dry skim milk in T-TBS (10 mM Tris, 150 mM NaCl, 0.1% Tween-20, pH 7.5). Then the membranes were incubated overnight at 4°C with a mouse monoclonal antibody that recognized σ_1 receptor (1:1,000, sc-137075, Santa Cruz Biotechnology, Dallas, TX, USA). Mouse monoclonal anti-β-actin antibody (1:5,000, sc-81178, Santa Cruz Biotechnology) was used as a loading control. Both primary antibodies were diluted in T-TBS containing 0.5% dry skim milk. The membranes were washed (3 × 10 min) with T-TBS and incubated for 1 h at room temperature with horseradish peroxidase-linked goat anti-mouse IgG (sc-2005, Santa Cruz Biotechnology), diluted to 1:2,500 in T-TBS containing 0.5% dry skim milk. The membranes were then washed (3 × 10 min) with T-TBS, and the bands visualized with an enhanced chemiluminescence method (ECL Prime Western Blotting Detection Reagents, Amersham Biosciences, Little Chalfont, Buckinghamshire, UK) according to the manufacturer's instructions (5 min incubation at room temperature). The ImageJ v1.49 program (National Institutes of Health, Bethesda, MD, USA) was used for densitometric analysis of the immunoreactive bands. The data are presented as the ratio of the intensity of the σ_1 receptor bands to the β-actin bands. To estimate the molecular weight of the resulting immunoreactive bands, we included in the same gel used for the

samples a mixture of 10 blue-stained recombinant proteins of different molecular weights (Precision Plus Protein All Blue Standards, Bio-Rad).

Using this procedure we identify 3 intense immunoreactive bands located at ~28 kDa, between 37-50 kDa, and at ~75kDa. We have previously reported that the immunoreactive bands located at ~28 kDa unequivocally correspond to σ_1 receptors, since they are not present in samples from σ_1 KO animals or in wild-type mice samples when the assay was performed in the absence of anti-σ_1 receptor antibody (4). The immunoreactive band between the 37 and 50 kDa landmarks corresponds to β-actin (molecular weight 43 kDa). Finally, the upper band, located near the 75kDa landmark, together to other weaker immunoreactive bands located below this 75 kDa band (see Supplementary Figure 2 for representative immunoblot images) are due to unspecific labeling, since they are present in σ_1 KO animals or in wild-type mice despite the omission of the anti-σ_1 receptor antibody in the procedure (data not shown).

2. Supplementary Figures and Tables

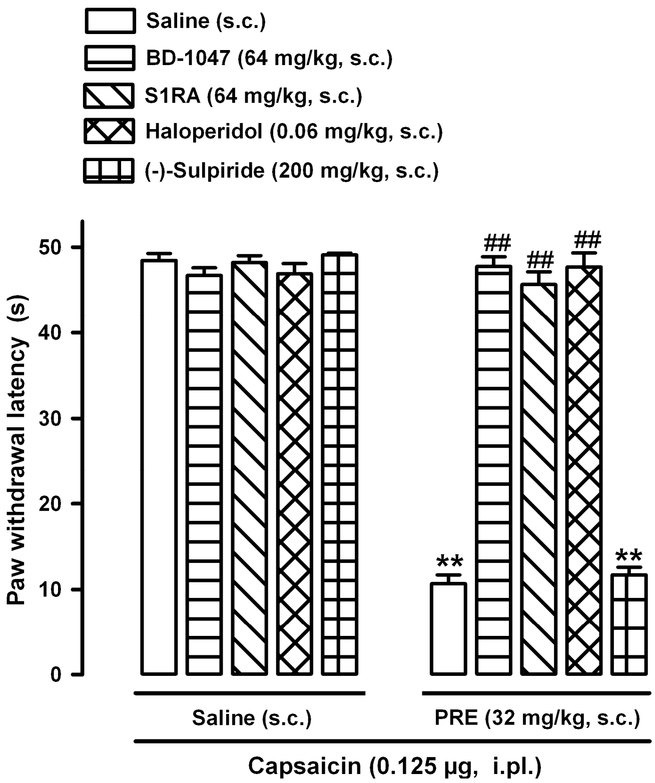

Supplementary Figure S1. Reversion of the effects of the σ_1 agonist PRE-084 on capsaicin-induced secondary mechanical allodynia by the σ_1 antagonists BD-1047, S1RA and haloperidol, and the dopaminergic antagonist (−)-sulpiride in wild-type mice. Wild-type mice were injected subcutaneously (s.c.) with BD-1047 (64 mg/kg), S1RA (64 mg/kg), haloperidol (0.06 mg/kg) and (−)-sulpiride (200 mg/kg) or their solvents (saline or 5% gum arabic) alone or associated with PRE-084 (PRE) (32 mg/kg). The solvent of haloperidol and (−)-sulpiride (5% gum arabic) did not alter the responses to mechanical stimuli in mice treated with either capsaicin or its vehicle (data not shown). The drugs tested here were administered 30 min before the intraplantar (i.pl.) injection of capsaicin (0.125 µg). The results represent the latency to paw withdrawal in response to stimulation with a punctate mechanical stimulus at 0.5 g force 15 min after capsaicin administration. The animals were always stimulated in the injected paw. Each bar and vertical line represent the mean ± SEM of the values obtained in 8–10 animals. The

difference between the values obtained in mice treated with the σ_1 agonist compared to the solvent-treated group was statistically significant at **p<0.01. The difference between the values obtained in mice treated with the σ_1 agonist alone or associated with each tested σ_1 antagonist was statistically significant at ##p<0.01. There were no statistically significant differences between the values obtained in mice treated with each antagonist alone compared to its solvent (two-way ANOVA followed by Bonferroni test).

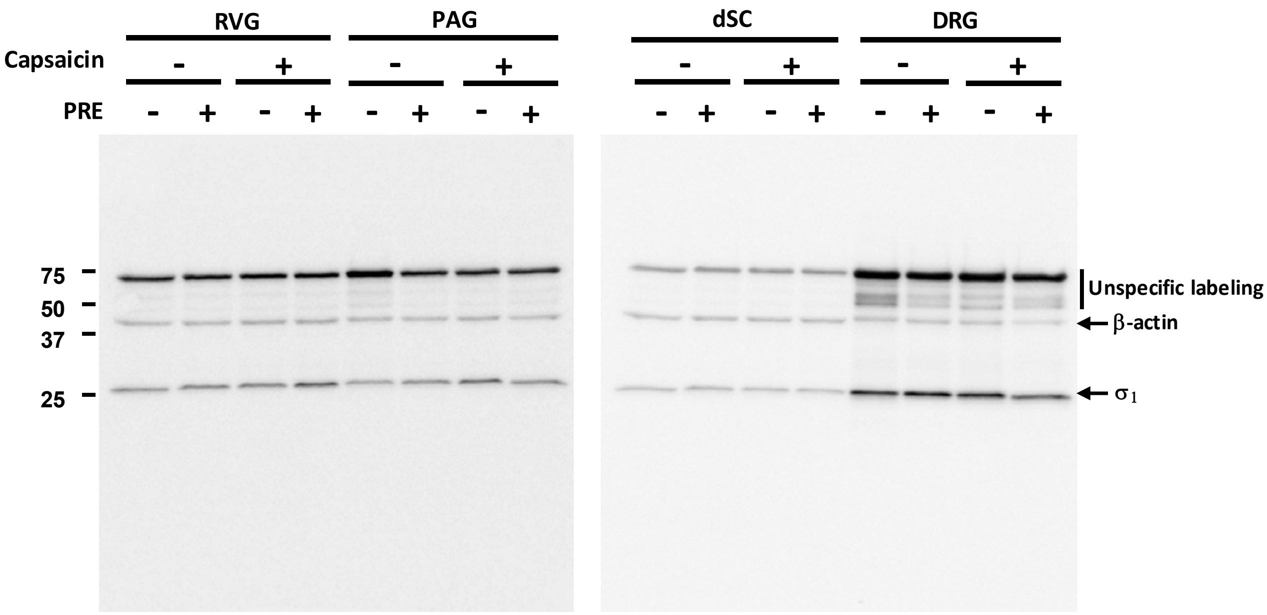

Supplementary Figure S2. Full-length gel of representative immunoblots for σ_1 receptors in mice after different treatments. Expression of σ_1 receptors in the rostroventral medulla (RVM), periaqueductal grey matter (PAG), dorsal spinal cord (dSC) and dorsal root ganglion (DRG) after the repeated administration of PRE-084 (PRE) in capsaicin-sensitized mice and nonsensitized animals. Mice were subcutaneously (s.c.) treated with PRE or its solvent (saline) once a day (q.d.) for 7 days. 24 h after the last s.c. injection, the mice were given an intraplantar injection of capsaicin (0.125 µg) or its vehicle (DMSO, 1%), and tissue samples were obtained 15 min later. β-actin was used as a loading control. We have previously shown that only the ~28 kDa band disappears in σ_1 KO animals or after omission of the anti-σ_1 receptor antibody.

Supplementary Table S1. *In vitro* metabolic stability of PRE-084 and pregabalin in mouse and human liver microsomes

Drug	Cl int (μL/min/mg protein)	$t_{1/2}$ (min)	% parent compound remaining at 1 h
PRE-084			
Mouse	949 ± 203	3 ± 0.67	0
Human	103 ± 5	13 ± 0.58	4 ± 0.58
Pregabalin			
Mouse	4.5 ± 1.2	602 ± 182	97 ± 2.67
Human	2.3 ± 0.94	1076 ± 646	95 ± 2.96

The % of the parent compound remaining at 1 h was determined by ultra-high performance liquid chromatography–tandem mass spectrometry. The *in vitro* metabolic half-life ($t_{1/2}$) and the intrinsic clearance (Cl int) were estimated from the Ln-linear plots of the % of compound remaining over time (see Methods for details). The data represent the mean ± SEM of three different experiments.

3. References

1. Cobos, E. J., Lucena, G., Baeyens, J. M., & Del Pozo, E. Differences in the allosteric modulation by phenytoin of the binding properties of the σ_1 ligands [^{3}H](+)-pentazocine and [^{3}H]NE-100. Synapse 59, 152-161 (2006)
2. Entrena, J. M. et al. Antagonism by haloperidol and its metabolites of mechanical hypersensitivity induced by intraplantar capsaicin in mice: role of sigma-1 receptors. Psychopharmacology (Berl) 205, 21-33 (2009).
3. Entrena, J. M. et al. Sigma-1 receptors are essential for capsaicin-induced mechanical hypersensitivity: studies with selective sigma-1 ligands and sigma-1 knockout mice. Pain 143, 252-261 (2009).
4. Sanchez-Fernandez, C. et al. Modulation of peripheral μ-opioid analgesia by σ_1 receptors. J. Pharmacol. Exp. Ther. 348, 32-45 (2014).
